# Annotations

**Published:** 1897-01-16

**Authors:** 


### ANNOTATIONS.

### The Queen's Reign.

THE authoritative statement made by the Prince of Wales for guidance as to the selection of the most fitting scheme by which the sixtieth anniversary of the Queen's accession may be celebrated, is as important as it is tactful. Two points are worthy of notice. First, that the Queen will readily appreciate any undertaking to celebrate the event which may be the outcome of the wish of the people, whether generally or locally expressed. We interpret this to mean that Her Majesty recognises that, all things considered, her reign can most usefully be commemorated by the inhabitants of each city and county selecting the particular object which is best calculated to promote the welfare of its inhabitants. Secondly, the belief expressed that due support will be given to works of mercy among the sick and suffering, or which may tend to brighten the lives and ameliorate the condition of Her Majesty's poorer subjects, testifies to the Queen's desire that every object shall possess distinct practical utility. We feel confident that the Prince of Wales' announcement, on authority, will put courage and heart into the supporters of the great voluntary charities of this country. They may now look forward with certainty to public opinion so shaping itself as to secure that the hospitals and medical institutions, as well as the great general charities, will not be forgotten when London and other great centres of population finally determine the form of commemoration for which public support will be invited.

### The Disinfection of Ships.

THE cleansing and disinfection of a ship and all its passengers is a matter of no small difficulty, and we may opine that in too many cases it is done in a more or less perfunctory manner. In fact, it is not easy to see how the officials of an emigrant ship with the ordinary appliances they have on board could satisfactorily accomplish the task. The want of properly arranged baths and of a steam disinfector tend to reduce the whole performance to one of mere fumigation, which, so far as the passengers are concerned, is a mere farce. In New York, however, the authorities have tackled the question in a thoroughly practical spirit. They have fitted up a steamboat with baths and disinfecting chambers, so that the passengers entering the bath-rooms at one end pass out at the other, where they receive their clothing, which has meanwhile been through the steam disinfector. The boat is also fitted with perchloride tanks and soda tanks, each with separate pump and hose systems, and a sulphur furnace with air pumps and reservoir. The boat with its staff of disinfectors is run alongside any ship which has to be dealt with, the passengers, their bedding, and their belongings are transferred, and while they are undergoing the process of bathing and steaming the whole ship is cleaned down and fumigated. For this purpose the soda solution is first used to remove grease, and render accessible surfaces which are in constant use; these are then washed down with the perchloride solution, which is followed by a thorough rinsing of sea-water. The sulphur furnace has four pans, each large enough to contain a couple of pails of sulphur, and the fumes are collected and drawn off as needed by a rotatory fan, and distributed through a system of supply pipes to the different parts of the vessel to be disinfected. The clean passengers then return to the clean ship. We understand that this disinfecting boat is already in commission, and ready for work, and that the tests which have been applied have proved very satisfactory. We cannot but feel that it is a move in the right direction.

### Soldiers' Typhoid in India.

SEVERAL "heartbroken parents" have recently published letters in the daily newspapers asking for the sympathy of the public in the griefs they have been called upon to suffer in consequence of the deaths of their sons, young military officers, from typhoid in India. It is not difficult to understand that whilst a a parent may hear of the death of a son in action against the enemies of his country almost without a pang, he may yet endure an agony of sorrow at the thought of the young man's long sufferings and lingering death from what so many of us know to be a preventable disease. Undoubtedly typhoid is preventable, and preventable in India. Nevertheless, in that country of old customs, base superstitions, and uneducated and conscienceless populations, the practical difficulty of preventing typhoid is so great as almost to be superhuman. The Annual Report for 1895 of the Chemical Examiner and Bacteriologist to the Governments of the north-west provinces and Oudh, and of the central provinces, has been recently published, and many of the statements contained therein make it almost hopeless to expect any marked improvement in health conditions, except what must be of very slow development indeed. The wells, of course, in the villages and elsewhere, are among the principal sources of danger; and if it be asked why new wells are not constructed from time to time, the answer must be that when they are they are as certain to be contaminated in no long time as those previously in use. In India "the trail of the native" is over everything; and the native does not, and will not understand that contaminated water, whether it is used for drinking, or for cooking, or for washing and cleansing purposes, may contain the germs of typhoid fever, and may kill as certainly as the imbibition of a known deadly poison. Efforts are constantly being made to purify drinking water, and all other waters which are used for any domestic purpose. Mr. Hankin, whose name is so well-known in this connection, thinks he has had a good deal of success in using permanganate of potash. But the difficulties, even in this case, of ascertaining for certain what results of a practical kind accrue, are almost insurmountable. With a population reckoned by hundreds of millions, and a few scientists whose numbers may almost be counted on the fingers of the two hands, it is to be feared that we must make up our minds that the risks to our young officers and privates in India from typhoid fever will not be much lessened for many years to come, unless, indeed, the youthful soldiers can be taught to take intelligent scientific precautions on their own account. In that case a much better state of things might reasonably be looked for within a very short period of time.

